# Preclinical Safety Assessment of the EBS-LASV Vaccine Candidate against Lassa Fever Virus

**DOI:** 10.3390/vaccines12080858

**Published:** 2024-07-30

**Authors:** Demetrius Matassov, Lisa Evans DeWald, Stefan Hamm, Rebecca M. Nowak, Cheryl S. Gerardi, Theresa E. Latham, Rong Xu, Amara Luckay, Tracy Chen, Marc Tremblay, Jeffry Shearer, Melissa Wynn, John H. Eldridge, Kelly Warfield, Kevin Spurgers

**Affiliations:** 1 Auro Vaccines LLC, Pearl River, NY 10965, USA; shamm@aurovaccines.com (S.H.); bnowak@aurovaccines.com (R.M.N.); cgerardi@aurovaccines.com (C.S.G.);; 2 Emergent BioSolutions Inc., Gaithersburg, MA 20879, USA; shearerj@ebsi.com (J.S.); wynnm@ebsi.com (M.W.); warfieldk@ebsi.com (K.W.); spurgersk@ebsi.com (K.S.)

**Keywords:** Lassa virus, LASV, glycoprotein, vesicular stomatitis virus, VSV, attenuated vaccine, biodistribution, preclinical safety, neurovirulence

## Abstract

There are currently no prophylactic vaccines licensed to protect against Lassa fever caused by Lassa virus (LASV) infection. The Emergent BioSolutions (EBS) vaccine candidate, EBS-LASV, is being developed for the prevention of Lassa fever. EBS-LASV is a live-attenuated recombinant Vesicular Stomatitis Virus (rVSV)-vectored vaccine encoding the surface glycoprotein complex (GPC) from LASV and has two attenuating vector modifications: a gene shuffle of the VSV N gene and a deletion of the VSV G gene. Preclinical studies were performed to evaluate EBS-LASV’s neurovirulence potential following intracranial (IC) injection and to determine the biodistribution and vector replication following intramuscular (IM) inoculation in mice. In addition, the potential EBS-LASV toxicity was assessed using repeated-dose IM EBS-LASV administration to rabbits. All mice receiving the IC injection of EBS-LASV survived, while mice administered the unattenuated control vector did not. The vaccine was only detected in the muscle at the injection site, draining lymph nodes, and the spleen over the first week following IM EBS-LASV injection in mice, with no detectable plasma viremia. No toxicity was observed in rabbits receiving a three-dose regimen of EBS-LASV. These studies demonstrate that EBS-LASV is safe when administered to animals and supported a first-in-human dose-escalation, safety, and immunogenicity clinical study.

## 1. Introduction

Lassa fever is an acute viral illness caused by the Lassa virus (LASV), a zoonotic, single-stranded, enveloped ribonucleic acid (RNA) virus in the family *Arenaviridae* [1] that can manifest as a severe viral hemorrhagic fever (VHF). Lassa virus has been documented across sub-Saharan West Africa and is endemic in Nigeria and countries in the Mano River region (Sierra Leone, Liberia, and Guinea) [2]. The disease is associated with significant morbidity and mortality as well as considerable economic and health security consequences [3]. It is estimated that there are about 300,000 to 500,000 Lassa virus infections per year, although an ongoing, large-scale Lassa epidemiology study in West Africa may help refine these estimates and provide a more accurate understanding of the LASV prevalence in that region (manuscript in preparation). While Lassa virus infections are asymptomatic in almost 80% of infected people, Lassa fever results in approximately 5000 deaths each year. The overall case fatality rate for Lassa virus infections is about 1%, while the case fatality rate for hospitalized patients with severe Lassa fever is around 15% [3,4]. Survivors of Lassa fever can experience long-term neurological sequalae, including hearing loss. Women who become infected during the third trimester of pregnancy are at a higher risk of severe outcomes; maternal death or fetal loss occur in up to 80% of these cases [1,5]. There are no approved therapeutic or prophylactic vaccines for the prevention of Lassa virus infection or Lassa fever. An effective prophylactic LASV vaccine would find application in endemic areas as well as with medical personnel and close contacts during natural outbreaks. Thus, Emergent BioSolutions (EBS) (Gaithersburg, MD, USA) advanced the development of the rVSV-N4ΔG-LASV-GPC1 vaccine candidate EBS-LASV, initially designed and developed by Profectus BioSciences Inc. (now Auro Vaccines LLC) (New York, NY, USA), for the prevention of disease caused by Lassa virus infection.

Vesicular Stomatitis Virus is a negative-sense RNA virus of the *Rhabdoviridae* family that is used as a vaccine vector against microbial pathogens [6]. The advantages of recombinant Vesicular Stomatitis Virus (rVSV)-vectored vaccines include fast production in approved, continuous mammalian cell lines such as Vero cells and the low VSV seroprevalence in the human population [6]. In a clinical trial of an attenuated rVSV-vectored human immunodeficiency virus (HIV) vaccine candidate, 0/60 (0.0%) subjects recruited at multiple sites across the United States (U.S.) were seropositive for VSV exposure (HVTN 090, www.ClinicalTrials.gov Identifier: NCT01438606 [7]). As VSV is not endemic in Africa, seroprevalence in African populations is expected to be low or non-existent, making the vector practicable for development as an LASV vaccine candidate [6]. The transcription of the VSV genome produces a gradient of mRNAs that serves to regulate the relative abundance of the corresponding VSV protein products in the order N > P > M > G > L. Disruption of the native gene order (i.e., by gene shuffling) can be used to alter the relative ratio of expressed virus proteins, resulting in the attenuation of the virus [8]. The ability to manipulate the genome to express a transgene of interest (i.e., vaccine antigen) and ellicit a robust immune response against the protein encoded by the transgene makes the rVSV-vector platform appealing for vaccine development [6]. rVSV-vectored HIV and Ebola virus vaccines that were attenuated using this approach were shown to be safe and immunogenic in Phase 1 clinical trials [6,9,10]. Another Ebola virus vaccine engineered on a wild-type rVSV backbone has been safely administered to several thousand subjects in West Africa [11]. However, vaccine-associated arthritis and rashes were observed with this non-gene-shuffled vaccine backbone [11,12,13]. This vaccine, developed by Merck (Rahway, NJ, USA), is currently registered by the trade name ERVEBO^®^ by several African National Health Authorities in the Democratic Republic of the Congo (DRC), Burundi, Ghana, and Zambia and has received approval from the US Food and Drug Administration (FDA) and European Medicines Agency (EMA) [12,13].

The neurotropism of wild-type VSV facilitated by the VSV glycoprotein (G) can lead to the infection of the central nervous system (CNS), causing neurological dysfunction and lethal paralysis [14]. The neurovirulent properties of VSV have been demonstrated in mice [15] and NHPs [16], where mice have been shown to be susceptible to fatal encephalitis after intracranial (IC) or intranasal (IN) inoculation with wild-type VSV [17]. With the neurovirulent (NV) nature of VSV, it is important for VSV-based vaccine candidates to be tested for any NV-associated pathogenesis [18]. The EBS-LASV vaccine candidate replaces the VSV glycoprotein (VSV G) with the LASV glycoprotein complex (GPC), thus potentially eliminating the NV nature of the virus. The NV potential of the EBS-LASV vaccine candidate was assessed in a mouse NV model, since mice are highly sensitive to VSV infection via IC inoculation, are readily available, and are a more cost-effective model than NHPs [8,19].

The safety of the EBS-LASV vaccine candidate expressing the LASV GPC has not previously been evaluated in preclinical studies. To support regulatory approval of a first-in-human clinical study, preclinical assessments were performed to evaluate the neurovirulence, biodistribution, and repeat-dose toxicity of the EBS-LASV vaccine. The assessments in these preclinical studies were designed to identify any potential or unknown systemic or organ-specific toxicity and to support the selection of safe vaccination regimens for human studies. Here, we describe the results from three preclinical studies designed to evaluate (1) the neurovirulence potential of EBS-LASV in young mice following intracranial (IC) injection, (2) the extent of EBS-LASV replication and biodistribution following intramuscular (IM) inoculation in mice, and (3) the potential toxicity of EBS-LASV following repeat-dose IM administration to rabbits. Strains of wild-type mice have been found to be resistant to LASV infection via many routes of exposure except for infection via intracranial (IC) inoculation, which leads to LASV disease and death [20]. Mice were selected as the animal model for the neurovirulence study, as young mice have been shown to be particularly sensitive to wild-type VSV infection when inoculated intracranially, succumbing to infection within two days [21,22,23]. The results of these preclinical studies supported the initiation of a Phase 1 clinical study by evaluating the safety and immunogenicity of EBS-LASV.

## 2. Materials and Methods

### 2.1. Generation and Characterization of EBS-LASV Vaccine Candidate

As previously described [24], EBS-LASV (also referred to as rVSV-N4ΔG-LASV-GPC1 or rVSV-Arena-074) is a live-attenuated, rVSV-vectored monovalent prophylactic vaccine that expresses the LASV GPC on the virion surface. For this work, the nucleotide sequence of LASV glycoprotein gene (Josiah strain; GenBank accession no. J04324.1) [25] was codon optimized to enhance the gene expression in mammalian cells and was de novo synthesized by Genewiz (South Plainfield, NJ, USA).

**Figure 1:**
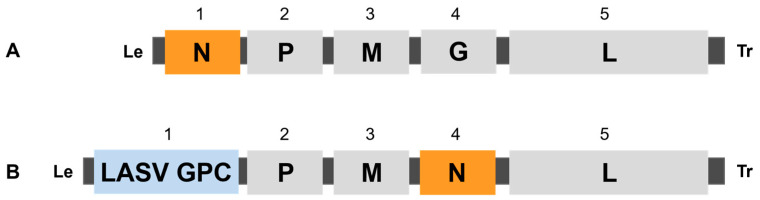
Genome organization of attenuated rVSV LASV vaccine candidate. (**A**) Organization of the wild-type VSV genome showing the nucleocapsid protein (N), phosphoprotein (P), matrix protein (M), glycoprotein (G), and large protein (L). The numbers indicate the genomic position of the transcriptional unit containing the individual open reading frame. The virus leader (Le), trailer (Tr), and gene junctions are shown in black. (**B**) Genome organization of the EBS-LASV vaccine candidate [rVSV-N4ΔG-LASV-GPC1]. The VSV N gene was shuffled to the 4th position (N4), the Lassa glycoprotein complex (GPC) gene was placed in the first position (GPC1), and the transcriptional cassette containing the VSV G gene was deleted from the virus genome (ΔG).

The EBS-LASV vaccine candidate was constructed by Auro Vaccines LLC (formerly Profectus BioSciences Inc.) with several genetic modifications to ensure vector attenuation, stability, and the robust expression of the LASV GPC transgene. EBS-LASV expressing LASV GPC from the first position in an rVSV-N4ΔG genome was generated by cloning the full-length LASV GPC gene into a multiple cloning site (MCS) within the first transcriptional cassette using XhoI/NotI restriction sites. (Figure 1). The VSV N gene was translocated to position 4 (N4), and the VSV G gene was deleted from the rVSV genome.

The EBS-LASV vaccine candidate was rescued from genomic cDNA as previously described [26]. The rescued virus supernatant was biologically cloned by two successive plaque isolations in 6-well plates and passaged nine times in T25 flasks under serum-free and antibiotic-free conditions using Vero cells (in-house traceable cell bank derived from ATCC CCL-81.2) adapted to grow in VP-SFM medium (Gibco, catalog no. 11681020) (Waltham, MA, USA). The amplified virus was further expanded at 32 °C on the same Vero cells grown in T-150 flasks at a multiplicity of infection (MOI) of 0.001. The culture medium was harvested, supplemented with 10X sucrose/phosphate buffer (10X SP, 75% sucrose *w*/*v*, 12.5 g/L K_2_HPO_4_, and 5.17 g/L KH_2_PO_4_, pH 7.4) as a virus stabilizer, clarified using low-speed centrifugation (3000× *g*, 10 min at 4 °C), and pooled. The virus aliquots, designated as Research Virus Seed (RVS), were flash frozen using an ethanol/dry-ice bath and stored at ≤−80 °C.

The EBS-LASV RVS was titrated using a 6-well plaque assay and characterized using full genomic sequencing to ensure that the attenuating mutations were intact and that no deleterious mutations were introduced into the LASV GPC gene; the expression of the LASV GPC gene was also confirmed using Western blotting and plaque immunostaining using a rabbit LASV GPC-specific polyclonal antibody (IBT, catalog no. 0307-001) (Rockville, MD, USA). Further testing of the EBS-LASV RVS was conducted by Charles River Laboratories (Malvern, PA, USA) for sterility (GP-V660.1), mycoplasma (agar cultivable, GP-V611.20 and RT-PCR, GP-V900.1), and bacteriostasis and fungistasis (GP-V717.1).

### 2.2. Test Article and Test Article Preparation

The test article material for each study was derived from formulated bulk drug-substance (DS) material generated by Auro Vaccines LLC under non-GLP and non-GMP laboratory conditions. The manufacturing and analytical methods followed Standard Operating Procedures (SOPs) with increased traceability of raw materials and the facilitation of Good Documentation Practices (GDPs). Briefly, a frozen vial of Vero cells (in-house traceable cell bank derived from ATCC CCL-81.2) adapted to the OptiPRO serum-free medium (Gibco, catalog no. 12309-019) was expanded into CellStack10 (CS10) vessels at 37 °C in 5% CO_2_. The Vero cells from the CS10s were inoculated with a target seeding density of ≥5 × 10^5^ cells/mL in a 15 L Applikon stirred bioreactor system and grown on Cytodex 1 microcarrier beads (GE Healthcare, catalog no. 17-0448-03) (Bengaluru, India) in 8 L of OptiPRO serum-free medium. The microcarrier beads with the Vero cells were agitated at 60 RPM at 37 °C with a dissolved oxygen (DO) set point of 35% under fed-batch conditions (half culture volume replaced per day). Three days post-inoculation, when the culture reached a cell density of 2.11 × 10^6^ cells/mL, the Vero cells were infected with the EBS-LASV RVS at an MOI of 0.001, and the vessel temperature was reduced from 37 °C to 32 °C. The culture was sampled daily to monitor the cytopathic effect (CPE) in the culture. With the peak CPE (consisting of ≥90% rounded or floating Vero cells) occurring 72 h post-infection (hpi), the culture medium was aseptically removed from the bioreactor, and the unprocessed harvest material (UHM) was conditioned by the addition of concentrated sucrose/phosphate/glutamic acid cryopreserving buffer (SPG; 7.5% sucrose *w*/*v*, 1.25 g/L K_2_HPO_4_, 0.517 g/L KH_2_PO_4_, and 1.02 g/L glutamic acid, pH 7.4). The conditioned UHM was clarified by passing it through a Sartopure PP2 8 μm filter (Sartorius, catalog no. 5595301P7) (Mumbai, India) and a Sartoclean CA 3 + 0.8 μm filter (Sartorius, catalog no. 5625304E7) train at room temperature. The harvest was pumped through the filters at a flow rate of 100 mL/minute. Subsequently, the clarified, conditioned bulk harvest was treated with 50 units per mL of Benzonase enzyme (EMD Millipore, catalog no. 1016950001) (Burlington, VT, USA) overnight at 4 °C. A 1 M MgCl_2_ stock was added to the harvest for a final target concentration of 1 mM MgCl_2_. Approximately 4 L of clarified, conditioned bulk harvest material was purified using ultracentrifugation at 28,000 rpm (SW28 rotor) with an Optima LK90 ultracentrifuge (Beckman Coulter) (Brea, CA, USA) for 90 min at 4 °C using a 10% sucrose cushion. The EBS-LASV pellets were resuspended with SPG buffer, and the sucrose-purified bulk concentrate material (drug substance, DS) was aliquoted into cryovials, flash frozen in a dry-ice/alcohol bath, and stored at ≤−65 °C. EBS-LASV DS material was titrated using a 6-well plaque assay and characterized using full genomic nucleotide sequencing, Western blotting and immunoplaque staining of the LASV GPC antigen, the residual host DNA (Quant-iT PicoGreen dsDNA Assay Kit, Invitrogen, catalog no. P7589) (Waltham, MA, USA), and the total protein (Pierce BCA Protein assay kit, ThermoFisher, catalog no. 23227) (Waltham, MA, USA).

For the neurovirulence study, the EBS-LASV test article was thawed rapidly immediately prior to use, and 10-fold serial dilutions ranging from 1 × 10^3^ to 1 × 10^7^ plaque-forming unit (PFU) were prepared in phosphate-buffered saline. The biodistribution study used the EBS-LASV test article at a concentration of 1 × 10^7^ PFU per 50 μL in SPG buffer.

For the GLP repeat-dose toxicology study, the EBS-LASV test article was formulated with SPG + gelatin (0.2% gelatin *w*/*v* buffer; Gelita, Sergeant Bluff, IA, USA, VacciPro^®^) and filled aseptically into 3 mL clear Type 1 glass vials (FIOLAX^®^, Schott North America, catalog no. 6896-0316) with Fluorotec-coated butyl rubber stoppers (West Pharmaceuticals, catalog no. 19700034) (Exton, PA, USA) at a concentration of 10.5 × 10^7^ PFU/mL; it was then stored at −80 °C and protected from light. Test article dose analysis was conducted to confirm the identity, strength, purity, composition, and stability. The commercially available reference article (0.9% sodium chloride for injection) was supplied by Baxter and stored in a temperature-controlled area at 21 °C.

### 2.3. Animals

Female outbred Swiss Webster (Crl:CFW(SW)) and BALB/c mice procured from Charles River Laboratories (Wilmington, MA, USA) were used for the neurovirulence and biodistribution studies, respectively. The animals were randomly assigned to each study group. Swiss Webster mice of five to six weeks of age have previously been used to assess the neurovirulence potential of attenuated recombinant VSV vectors, since they are susceptible to infection via the intracranial route [8,19]. The mice were monitored daily post-injection for signs of clinical illness. BALB/c mice of six to seven weeks of age at the time of inoculation were previously used [27] and were chosen for the biodistribution study for their ease of handling (i.e., more docile than other strains), ability to be readily infected experimentally via a variety of inoculation routes, and for being a good model to assess the immunogenicity and dissemination of live-attenuated VSV-based vectors. For the mouse studies, five mice per cage were housed at ambient temperatures between 20 and 24 °C, provided with adequate air circulation and 12-h light/12-h dark cycles except when interrupted for designated procedures. The mice were checked daily for adequate food and bedding conditions, with water accessible at all times.

New Zealand White rabbits (NZW, *Oryctolagus cuniculus*) received from Charles River Canada Inc. (St. Constant, QC, Canada) were used for the IND-enabling N + 1 toxicology study. The rabbits were four months old and weighed between 2.8 and 3.9 kg upon the initiation of dosing. The NZW rabbit was chosen as the animal model, because it is an accepted non-rodent species for pre-clinical toxicity testing by regulatory agencies [28,29]. Each animal for this study was identified using a subcutaneously implanted electronic identification chip. A period of 13–14 days was allowed between animal receipt and the start of dosing to acclimate the animals to the laboratory environment. The animals were randomly assigned to groups. Males and females were randomized separately. The disposition of all animals was documented in the study records. Male rabbits were individually housed, and female rabbits were group housed (two of the same dosing group together) in stainless steel cages with a perforated floor equipped with an automatic watering valve. The animals were maintained at an ambient temperature between 17 °C and 23 °C, with a target humidity between 30% and 70% and a 12-h light/dark cycle. All rabbits had access to a standard certified pelleted commercial laboratory diet once daily. For psychological/environmental enrichment, the rabbits were provided with items such as a hiding device and a chewing object except when interrupted by study activities.

All research studies involving the use of animals were reviewed and approved by the Institutional Animal Care and Use Committee of Auro Vaccines LLC and Charles River Laboratories. The studies were carried out in strict accordance with the recommendations in the Guide for the Care and Use of Laboratory Animals [30] and the Canadian Council on Animal Care [31].

### 2.4. Mouse Neurovirulence Study

The animals were randomly assigned to groups and given unique identifier numbers. Groups consisting of 10 Swiss Webster mice each were anesthetized by intraperitoneal injection of a mixture of ketamine (100 mg/kg of body weight) and xylazine (5 mg/kg) and injected intracerebrally with a 0.02 mL dose of EBS-LASV (1 × 10^3^ to 1 × 10^7^ PFU/animal), neurovirulence positive control vector rVSV_IN_-HIVGag5 (1 × 10^2^ PFU/animal), or sterile PBS. The animals were monitored daily for signs of clinical illness following inoculation, with increased observations (at least three times daily at four-hour intervals) during the critical period: two to six days post-inoculation. Observations included appearance, food and water intakes, behavior, and clinical signs of increased cardiac or respiratory rates. The animals were monitored to Study Day 14 post-inoculation (study endpoint), and those exhibiting severe clinical signs of illness, including paralysis, or reaching 80% of their initial body weight during the 14-day observation period were humanely euthanized via CO_2_ inhalation.

### 2.5. Mouse Biodistribution Study

The biodistribution of the EBS-LASV vaccine candidate was evaluated by measuring the presence and level of the vaccine in organs and tissues following IM administration. Prior to the initiation of the biodistribution study, it was necessary to measure the lower limit of detection (LLOD) and lower limit of quantification (LLOQ) of the EBS-LASV vaccine in mouse plasma to determine the viremia level of the EBS-LASV vaccine and whether perfusion of the mouse organs was required before harvesting. If significant levels of the EBS-LASV vaccine were present in the mouse plasma, then the mice would need to be perfused with a sterile 0.9% saline solution to eliminate the vaccine from the blood vessels within the organs and tissues; otherwise, the additional virus would introduce bias in the biodistribution results. For viremia evaluation, a total of 20 female BALB/c mice were inoculated in the left calf muscle with a single 50 μL dose of EBS-LASV at 1 × 10^7^ PFU formulated in SPG buffer (0.02 M sucrose, 0.7 mM K_2_HPO_4_, 0.38 mM KH_2_PO_4_, and 0.5 mM glutamic acid, pH 7.4). Groups of five mice were euthanized via CO_2_ inhalation on Study Days 0, 1, 2, and 3 post-immunization. Whole blood was collected via a cardiac punch and added to lithium heparin-containing tubes. The whole blood was centrifuged for five minutes at 2000× *g*, and the plasma was collected, aliquoted, snap frozen in ethanol dry ice slurry, and stored at ≤−80 °C in advance of the plaque assay.

For vaccine detection in organs and tissue, a total of 36 BALB/c mice (18 females and 18 males) were inoculated in the left calf muscle with a single 50 μL dose of EBS-LASV at 1 × 10^7^ PFU formulated in SPG buffer. Groups of six mice (three males and three females) were then euthanized via CO_2_ inhalation on days 0, 1, 2, 3, 5, or 10 post-immunization. The viremia study indicated that perfusion was not necessary prior to harvesting the mouse organs, as the viremia level of EBS-LASV was below the limit of detection and would not impact the biodistribution results. From each mouse, the brain, kidneys, spleen, left lobe of the liver, lungs, left calf muscle (site of injection), draining lymph nodes (popliteal, inguinal, and iliac), and one testis or 2 ovaries/uterus were collected, weighed, and suspended in an appropriate volume of SPG buffer (10% weight/volume, *w*/*v*), followed by homogenization. The samples were centrifuged, and the resulting supernatants were aliquoted and snap frozen. These samples were titrated using a plaque assay for the detection of the EBS-LASV vaccine candidate. The samples were given unique identifier numbers so that the analysts performing the assay would be blinded. Due to the small size/weight of draining lymph nodes, additional SPG buffer was added to generate enough sample for testing; titers were normalized based on the actual percent *w*/*v*. The harvested organs and tissues were tested using a plaque assay to detect the presence and levels of the EBS-LASV vaccine candidate after vaccination. On the day of the plaque assay, the samples and positive control were quickly thawed in a 37 °C water bath and then stored on ice until titration.

### 2.6. Plaque Assay

Briefly, Vero cells (ATCC CCL-81) were first seeded in 6-well plates at a density of 5 × 10^5^ viable cells/well. After 24 ± 6 h, the samples were serially diluted 10-fold in Dulbecco’s Modified Minimal Eagle’s (DMEM) (Corning, catalog no. 10-017CM) growth medium supplemented with 10% fetal bovine serum (FBS) and added (0.1 mL) to the confluent cell monolayers. Culture plates were placed on a rocker for 15 min at room temperature and then incubated for 30 min in a 37 °C incubator. Following incubation, the medium was removed from each well, after which the wells were overlaid with 3.0 mL of 0.8% Sea Plaque agarose (Lonza, catalog no. 50100) (Basel, Switzerland) in DMEM medium supplemented with 10% FBS. The culture plates were incubated at 32 °C/5% CO_2_ and monitored for three to four days for plaque formation. When distinct plaques were visible, the agar overlays were removed, and each well was stained for 10–15 min with 0.5–1 mL of 0.5% crystal violet stain solution. After washing and drying the plates, the plaques were counted, and a titer (PFU/100 μL) was calculated. If no plaques were observed after seven days, the six-well plates were stained, and the plaque count was recorded as less than the LLOD.

The characterization of the plaque assay using mouse plasma, tissue, and organs spiked with varying amounts of a well-characterized stock of EBS-LASV (100, 20, 10, and 2 PFU/100 μL) was performed, and the LLOD and the LLOQ of the assay were determined prior to the biodistribution study. The LLOD was 2 PFU/100 µL, and the LLOQ was 100 PFU/100 µL for mouse plasma. The LLOD of the plaque assay was 1 PFU/10 mg tissue (10 PFU/100 μL) for all mouse organs. The LLOQ of the plaque assay was 2 PFU/10 mg tissue (20 PFU/100 µL) for all mouse organs except for the brain and liver. The brain and liver LLOQs were determined to be 20 PFU/10 mg tissue (200 PFU/100 µL).

### 2.7. GLP Repeat-Dose Rabbit Toxicology Study

#### 2.7.1. Experimental Design and Dose Administration

EBS-LASV was administered to male and female NZW rabbits on Study Days 1, 22, and 43 at 5.3 × 10^7^ PFU per dose, as indicated in Table 1. EBS-LASV and saline control material were administered as IM injections of 0.5 mL into the lumbar area. Prior to dosing, the fur was shaved or clipped around the lumbar area. After injection, the skin was marked with indelible ink for observation of the dosing sites and for tissue collection at necropsy. The area was clipped and marked as often as necessary throughout the study thereafter. The duration, reversibility, or delayed onset of any adverse effects were assessed following 3- and 28-day recovery periods after the administration of the final dose. Each treatment group was divided into two subgroups: Main Study and Recovery Study. Animals were euthanized at the scheduled study endpoint, either 3 days (Study Day 46) or 28 days (Study Day 71) after the final dose for the Main Study or Recovery Study groups, respectively. In-life and post-life procedures were performed to monitor for possible signs of toxicity in both the Main and Recovery Study animals. The study was performed in accordance with the U.S. Department of Health and Human Services, Food and Drug Administration, United States Code of Federal Regulations, Title 21, Part 58: Good Laboratory Practice for Nonclinical Laboratory Studies and as accepted by Regulatory Authorities throughout the European Union (OECD Principles of Good Laboratory Practice). Assay operators were blinded to animals receiving either the control or test article, with each animal provided with a unique subject ID number. The sample type and day of sample collection were visible to the assay operators.

#### 2.7.2. Clinical and Injection Site Observations

Cage side moribundity and mortality observations were performed twice daily, once in the morning and once in the afternoon. Detailed clinical observations were performed at least once during the pre-dosing period, whereas during the dosing period, clinical observations occurred weekly and at 2 to 4 h post-dose. During these observations, the injection site was observed for signs of erythema/edema prior to dosing and at 3, 24, 48 h (±2 h), and 72 h (±2 h) post-dosing and prior to the necropsy of Recovery Study animals. The observations were scored according to the modified Draize scoring scale. Rectal body temperature measurements were conducted prior to dosing, 2–4 h after dosing, and prior to the scheduled necropsies. Unfasted animals were weighed individually 1 week prior to the first dose, at 72 and 96 h (±2 h) after each dose, weekly during non-dosing weeks, and 2 days prior to each dose. A fasted weight was recorded on the day of necropsy. Food consumption was quantitatively measured daily starting on Study Day 7 and continuing throughout the dosing and recovery periods, except for the day of the scheduled euthanasia. Once during the pre-dosing period and at the end of the dosing and recovery periods, all animals were subjected to funduscopic (indirect ophthalmoscopy) and biomicroscopic (slit lamp) examinations. The mydriatic drug used was 1% tropicamide.

#### 2.7.3. Clinical Pathology

Blood for clinical pathology [hematology, clinhical chemistry, and coagulation (except fibrinogen)] was collected from the auricular veins of all animals 7 days prior to the first dose, on Study Days 3, 24, and 45, and, in addition, on Study Day 71 for animals from the Recovery Group. Separate blood samples for fibrinogen analysis were collected pre-dosing (Study Days 1, 22, and 43) and 7 days post-dosing (Study Days 8, 29, and 50). The animals were fasted overnight before blood sampling (for clinical chemistry) and urine collection (for urinalysis). Additional blood samples were obtained (due to clotting of non-serum samples) when permissible. After collection, the samples were transferred to the appropriate laboratory for processing. Hematology analyses were conducted on whole blood samples supplemented with K-EDTA anticoagulant using a Siemens ADVIA 120 Analyzer (GMI, Ramsey, NJ, USA). Blood smears were prepared from each hematology sample and read to investigate results, when necessary. Blood samples with no anticoagulant collected in serum separator tubes were processed to serum and analyzed for clinical chemistry parameters using a Modular Analytics/Cobas 6000 Analyzer (Roche, Basel, Switzerland). Blood samples for coagulation analyses were collected in tubes containing citrate anticoagulant, processed to plasma, then analyzed for coagulation factors using a Stago Compact Coagulation Analyzer (Asnières-sur-Seine, France). Urine samples were collected via cystocentesis at necropsy on Study Days 46 and 71 and analyzed using a Clinitek ATLAS (Forchheim, Germany) or NOVUS analyzer (Manchester, UK).

#### 2.7.4. C-Reactive Protein (CRP) Evaluation

Blood samples for CRP evaluation were collected in serum separator tubes from the auricular arteries/veins of all animals 7 days prior to the first dose, on Study Days 2, 3, 4, 23, 25, 44, and 46, and, in addition, on Study Day 71 for animals from the Recovery Group. The samples were allowed to clot at room temperature, after which they were processed to serum. The resulting serum was separated, transferred to clear polypropylene tubes, and stored at −20 °C. Analysis was conducted using a GLP-validated ELISA method developed by Charles River Laboratories Montreal for the detection of CRP in rabbit serum.

#### 2.7.5. Vaccine Shedding

Rabbit blood and saliva samples for viral shedding analysis were collected on Study Days 2, 4, 8, 14, 23, 25, 29, 35, 44, 46, 50, and 71. Blood samples were collected in sodium heparin tubes and processed to plasma. Saliva samples were collected using saliva collection tubes pre-filled with 25 μL of concentrated SPG buffer. Plasma and saliva samples were analyzed for the presence of EBS-LASV. Briefly, 24-well plates were seeded with Vero cells supplemented with Dulbecco’s modified Minimal Eagle’s medium (DMEM) + 10% FBS (DMEM10) at a cell density of 1 × 10^5^ cells per mL and incubated at 37 °C in a CO_2_ incubator overnight (24 h ± 6 h). On the following day, the growth medium was aspirated from the plates, and 100 μL of DMEM was added to each well. The rabbit biological fluid was added at 100 μL per well in duplicate wells. Both positive and negative control samples were included in duplicate on each plate. The plates were incubated at room temperature for 15 min followed by an incubation at 37 °C for 30 min in CO_2_ incubator. The inoculum was aspirated, and 1 mL of DMEM10 was added to all wells of the plates. The plates were incubated at 32 °C 5% CO_2_ for 4–7 days. Under the light microscope, the Vero cell monolayers were monitored daily for the presence of an EBS-LASV-induced CPE. If any abnormal cell morphology was seen as compared to the negative control wells, the supernatant from the well was transferred to a new well with a Vero cell monolayer for an additional four- to seven-day incubation period. If the CPE was observed again, the potential presence of EBS-LASV was verified by performing a plaque assay and via immunostaining of the virus plaques with an antibody specific for the LASV glycoprotein.

#### 2.7.6. Immunogenicity Evaluation

Blood samples for immunogenicity evaluation were collected in serum separator tubes from the auricular artery/vein once during study week 1 and prior to dosing on Study Days 22 and 43. The serum samples were analyzed using an ELISA for the quantification of antibodies to the LASV glycoprotein.

Immulon HB 96-well high-binding microtiter plates were coated overnight at 4 °C with a gamma-irradiated Lassa virus (NR-31822, BEI Resources) coating antigen. The plates were then washed 3 times with 300 µL/well of 0.1% Tween-20 in Dulbecco’s modified Phosphate-Buffered Saline (DPBS) and blocked for 2 h at room temperature in 5% non-fat dry milk with 0.1% Tween-20 in DPBS. Rabbit serum samples were diluted with 5% non-fat dry milk and 0.1% Tween-20 in DPBS, and 100 µL/well was added to the ELISA plates at a starting dilution of 1:100 and further diluted 3-fold across the plates. The plates were incubated at 4 °C overnight, after which they were washed 3 times with 300 µL/well of 0.1% Tween-20 in DPBS. A peroxidase-conjugated goat anti-rabbit IgG (Cat#11-035-008, Jackson ImmunoResearch Labs) was diluted to 1 in 10,000 with 5% non-fat dry milk and 0.1% Tween-20 in DPBS, and 100 µL/well was added for 1 h at room temperature. After 5 washes with 300 µL/well of 0.1% Tween-20 in DPBS, the plates were incubated for 1 h at room temperature with 100 µL/well of the peroxidase substrate, TMB (3,3′,5,5′-tetramethyl-benzidine, Cat#T-0440, Sigma). After 10 min at room temperature in the dark, the enzymatic reaction was stopped by the addition of 100 μL/well of 1 N Sulfuric Acid stop solution. The absorbance for each well was measured at 450 nm using a SpectraMax PC340 microplate reader (Molecular Devices). Data were evaluated using the SoftMax Pro v5.4 software (Molecular Devices) and Excel Workbook (Microsoft). LASV-specific serum IgG endpoint titers were defined as the reciprocal of the last serum dilution giving an optical density greater than 0.1 at 450 nm (OD_450_). The ELISA IgG endpoint titer was then exported to the Excel Workbook (Microsoft) for further statistical analysis. For the samples with an endpoint titer <100 (LOQ, limit of quantification), “50” was assigned for the statistical analysis.

Two control samples, Quality Control Low (QCL) and Quality Control High (QCH), as well as one negative control (NC) sample were utilized in the assay. The target range of anti-LASV IgG antibody endpoint titers for QCL was 2208–5199; for QCH, it was 43,961–81,664. The readout of NC OD_450_ was <0.1. This defined target range of anti-LASV IgG antibody titers for QCL and QCH and readout OD_450_ for NC were used as acceptance criteria during the testing of rabbit sera with unknown quantities of anti-LASV IgG antibodies.

The Main Study and Recovery Study animals were euthanized by intravenous sodium pentobarbital injection followed by exsanguination via an incision of the axillary or femoral artery on Study Days 46 and 71, respectively. The Main Study and Recovery Study animals were subjected to a complete necropsy examination that included the following: evaluations of the carcass and musculoskeletal system; all external surfaces and orifices; cranial cavity and external surfaces of the brain; and thoracic, abdominal, and pelvic cavities with their associated organs and tissues. The brain, epididymis, adrenal gland, pituitary gland, prostate gland, thyroid gland, heart, kidneys, liver, lung, ovary, spleen, testis, thymus, and uterus were weighed at weight ratio (using the terminal body weight), and the organ-to-brain weight ratios were calculated. The following organs were collected and preserved in 10% neutral-buffered formalin: adrenal glands, aorta, bone marrow (sternum), brain, esophagus, eyes, heart, large intestine, kidneys, liver, lungs with mainstem bronchi, lymph nodes (mesenteric and popliteal), mammary glands, optic nerves, ovaries (females), pancreas, pituitary, prostate (males), salivary glands, harderian gland, sciatic nerve, skeletal muscle (injection site and non-injected), small intestine, spinal cord, spleen, stomach, testes with epididymides (males), thymus, thyroid and parathyroid, trachea, urinary bladder, and uterus with cervix (females). Tissues (except bone marrow smears) were embedded in paraffin, sectioned, mounted on glass slides, and stained with hematoxylin and eosin. Histopathological evaluation was performed by a board-certified veterinary pathologist to assess any abnormalities or gross lesions in the collected tissues.

#### 2.7.7. Statistical Analysis

Statistical analyses were performed with an in-house reporting software, Nevis 2012, using the SAS 9.4 (SAS Institute Inc., Cary, NC, USA) statistical package. Levene’s test was used to assess the homogeneity of group variances with parametric assumption at the 5% significance level. The groups were compared using Dunnett’s test (equivalent to a *t*-test in Nevis 2012 tables) when Levene’s test was not significant or Dunn’s test (equivalent to the Wilcoxon Rank-Sum test in Nevis 2012 tables) when it was significant.

## 3. Results

### 3.1. Mouse Neurovirulence Study

The mouse neurovirulence testing results for EBS-LASV are shown in Table 2. Mice that received varying doses of up to 1 × 10^7^ PFU of EBS-LASV did not show any signs of clinical illness, and all survived to the end of the study. These results demonstrated that when compared to an rVSV vector with a wild-type genome backbone expressing a foreign transgene, the neurovirulence potential of EBS-LASV was eliminated through attenuation in this sensitive animal model. All mice in the positive control group succumbed or reached endpoint criteria for euthanasia before the end of the 14-day observation period.

### 3.2. Mouse Biodistribution Study

**Figure 2:**
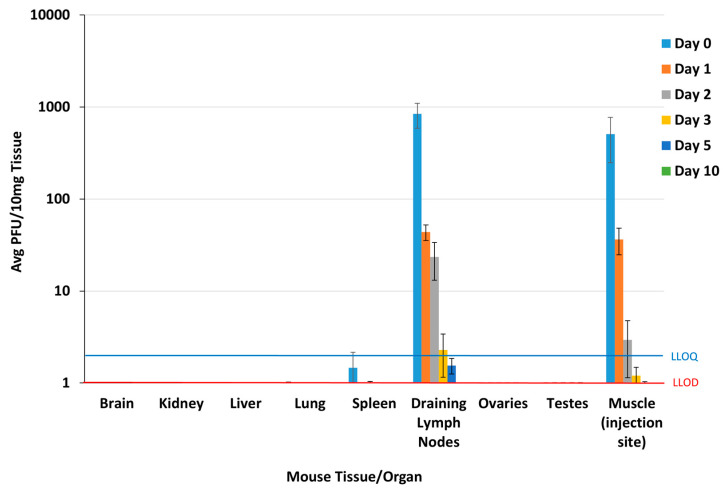
Plaque assay results from mouse biodistribution study. The LLOQs for the brain and liver were higher than depicted above; however, all levels were below the LLOD. LLOQ = lower limit of quantitation; LLOD = lower limit of detection.

Viremia was below the LLOD (2 PFU/100 µL) in all blood samples at all timepoints following a single IM injection of EBS-LASV at 1 × 10^7^ PFU/dose, demonstrating that EBS-LASV does not spread from the injection site into the blood in the murine animal model at this dosing regimen. EBS-LASV titers were at or below the LLOD (1 PFU/10 mg) in the brain, liver, kidney, lungs, ovaries/uterus, and testes on all harvest days (Figure 2). EBS-LASV levels were slightly above the LLOD in the spleen on Study Day 0 (1.9 ± 1.4 PFU/10 mg), ≤LLOD on Study Days 1 and 2, and below the LLOD in all mice by Study Day 3. EBS-LASV was present at detectable levels (above the LLOQ, 2 PFU/10 mg tissue) at the injection site (muscle) on Study Days 0, 1, and 2, with an average titer of 5 × 10^2^ PFU/10 mg on Study Day 0 and ≤10 PFU/10 mg on Study Day 1. This decreased to just above detectable levels (≤LLOD) by Study Day 5 and was undetectable by Study Day 10. The vaccine vector was present at detectable levels in the draining lymph nodes up to Study Day 3 but cleared to ≤LLOD by Study Day 10.

### 3.3. GLP Rabbit Toxicology Study

The administration of EBS-LASV did not result in early mortality or any changes in clinical observations (Table S1A–D). All clinical observations that occurred, including skin discoloration, scabbed skin, decreased activity, and vocalization, were considered unrelated to EBS-LASV, as the observations occurred at a similar incidence in the control group, in individual animals, and/or the observations were transient and did not persist.

**Figure 3:**
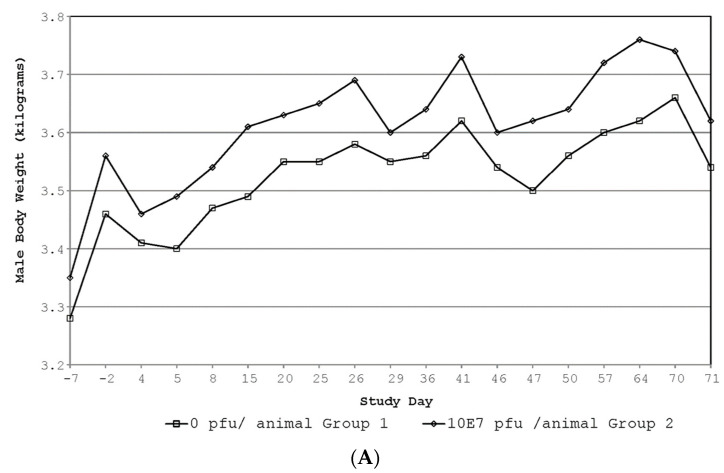

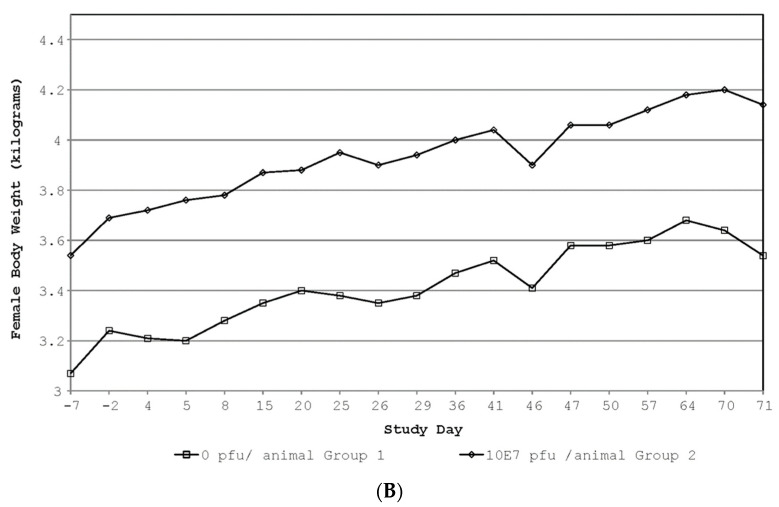
Rabbit body weight data. (**A**) Summary of male body weights; (**B**) summary of female body weights.

No EBS-LASV-related signs of erythema or edema at the injection sites, body temperature (Table 3), body weight (Figure 3), food consumption, ophthalmic, hematology, or clinical chemistry (Table S2A–D) changes occurred during this study. Any statistically significant or apparent differences in these parameters were considered not to be EBS-LASV-related due to one or more of the following: small magnitude of the difference, inconsistent direction of the difference across sexes, and general overlap in magnitude of individual values with controls or pre-dosing results.

Transient increases (1.31-fold greater than controls) in fibrinogen were noted in males on Study Day 3 (Table 4). These increases were not observed on subsequent timepoints or on any occasion for females. Following each day of dosing, transient increases in CRP were noted for males and females (Table 4) when compared to control animals. The day following each day of dosing (24 h post-dose), increases in CRP were noted and ranged between 614% and 1000% for males and 164% and 248% for females when compared to control. On Study Day 3, the increases were 982% and 64% for males and females, respectively, and returned to baseline on Study Day 4. On Study Days 25, 46, and 71, the results were comparable to controls and/or baseline values.

**Figure 4:**
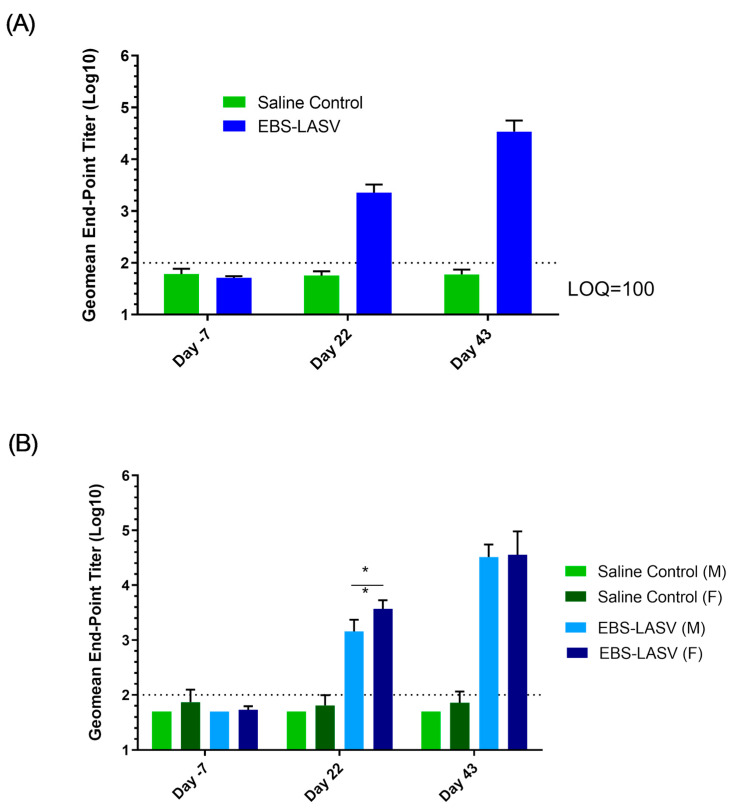
Rabbit serum LASV GP IgG endpoint titers (geomean ± 95%CI on Study Days −7, 22, and 43). (**A**) Geometric mean endpoint titers of saline control and EBS-LASV groups. (**B**) Geometric mean endpoint titers of male and female rabbits within each group. * *p* = 0.0054 at *t*-test.

All saliva and plasma samples analyzed for the shedding of EBS-LASV lacked EBS-LASV based on the CPE in Vero cell monolayers. All vaccinated animals seroconverted to EBS-LASV, as detected by an at least eight-fold LASV GP-specific IgG ELISA antibody titer increase at Study Days 22 and 43 compared to baseline (Figure 4). The geometric mean of the endpoint titer was greater than 3 and 4 log10 at Study Days 22 and 43, respectively, confirming that all animals received the test article and that EBS-LASV induced an LASV GP-specific immune response. By the rabbits eliciting a robust immune response against the EBS-LASV vaccine, it provides a way of assessing if any vaccine-related adverse effects (i.e., release of various pro-inflammatory cytokines) or toxicities will arise and be detected [32]. The stimulation of the cellular and/or the humoral immune system can potentially correlate to any adverse effects seen. For example, the EBS-LASV vaccinated male and female rabbits had transient increases in CRP, which is indicative of an inflammatory response, as compared to the control animals, thus showing that the EBS-LASV vaccine-stimulated immune response led to the transient CRP levels.

No EBS-LASV-related gross findings were noted, and there were no EBS-LASV-related alterations in the organ weights (Table 5). Some statistically significant differences were observed in the EBS-LASV vs. saline-treated group mean values at the end of the Main (Study Day 46) and Recovery (Study Day 71) Study groups. At the end of the Main Study, these included higher absolute spleen weights in males and higher absolute brain, kidney, liver, pituitary gland, and thyroid gland weights in females. At the end of the Recovery Study, these included higher absolute brain weights in males and higher absolute thymus and thyroid gland weights in females. The higher (brain, kidney, liver, and thyroid gland) organ weights in females receiving EBS-LASV were the consequence of higher terminal body weights in the Main (18%, when compared to saline controls) and Recovery Studies (17%). As for females receiving EBS-LASV, the pituitary gland mean organ weights relative to the body weight (Main Group) and the thymus mean organ weights relative to the body weight (Recovery Group) remained statistically higher than in the female saline control groups. However, the mean absolute pituitary gland weight (0.045 g) in EBS-LASV females was within the range for controls from the historical organ weight data (0.013 to 0.072 g) (Table S3). Only the thymus mean absolute organ weight (6.08 g) in EBS-LASV females was higher than the range for controls from the historical organ weight data (1.375 to 5.349 g). Females receiving EBS-LASV had statistically higher mean body weights as compared to the females receiving saline during the Main and Recovery Studies. However, any variations, including those that attained statistical significance, were within the range of the concurrent saline control group data and/or were considered to be due to biological variation. For males receiving EBS-LASV, there were no microscopic correlations to the higher spleen weights (Main Study) and brain weights (Recovery Study). The mean absolute spleen weight (1.40 g) in EBS-LASV males was comparable to the saline control group values from the historical organ weight data for the spleen (1.35 g). The higher mean brain weight was only observed in the EBS-LASV Recovery Group males and for the absolute organ weight mean. Also, the absolute brain weight mean value (10.73 g) was within the range for controls from the historical organ weight data (8.38 to 11.207 g).

A review of the gross necropsy observations revealed no observations that were considered associated with the administration of the EBS-LASV vaccine (Table S4). There were no EBS-LASV vaccine-related histologic changes in the dosing or recovery phase animals. All histologic changes were considered incidental findings or related to some aspect of experimental manipulation other than the administration of the EBS-LASV vaccine. There was no EBS-LASV vaccine-related change in the prevalence, severity, or histologic character of these incidental tissue alterations.

## 4. Discussion

Nonclinical neurovirulence, biodistribution, and repeat-dose toxicity studies were conducted to evaluate the safety of the EBS-LASV vaccine candidate prior to first use in humans. The neurovirulence study confirmed that the genetic modifications characteristic for this live-attenuated rVSV-based vaccine candidate ablated the inherent neurovirulence potential of wild-type VSV in mice, which are a particularly sensitive animal model for when VSV infection occurs intracranially [21,22,23]. Following IC infection with wild-type VSV, mice experience severe weight loss, develop bilateral hind-limb paralysis, and succumb to the infection within two days. The prototype lab-adapted recombinant VSV (Indiana) vector, rVSV-HIVGag5, expressing HIV *gag* from the fifth transcriptional cassette with no other modifications in the otherwise wild-type genetic backbone, is also highly lethal in mice after IC inoculation, with a similar LD50 to that of wild-type VSV (<5 PFU) [19]. The rVSV-HIVGag5 vector was used as a positive control, and differences in neurovirulence were determined between rVSV-HIVGag5 and EBS-LASV. Doses of up to the highest proposed clinical dose (1 × 10^7^ PFU) of EBS-LASV were safe following IC injection in mice, providing direct evidence that inherent neurovirulent properties of wild-type VSV were successfully removed in the rVSV-based EBS-LASV vaccine candidate due to engineered attenuation markers.

Biodistribution studies performed in mice by other researchers have demonstrated that when a live-attenuated, replication-competent rVSV vector is injected intramuscularly, it undergoes limited propagation at the injection site and in the local draining lymph nodes but does not spread to other organs and tissues [33]. EBS-LASV undergoes limited propagation at the injection site and in the local draining lymph nodes as well and does not spread at quantifiable levels to other organs and tissues. These results are consistent with those obtained for other previously described highly attenuated rVSV-based vaccine vectors [33,34] and thereby demonstrate a key safety feature for the EBS-LASV vaccine candidate.

The repeat-dose toxicology study assessed the potential toxicity of EBS-LASV when administered intramuscularly to New Zealand White rabbits once every 3 weeks (N + 1 dose) at a dose at least as high as the maximal intended clinical dose. The duration, reversibility, or delayed onset of any adverse effects was assessed following 3- and 28-day recovery periods after the administration of the final dose. The EBS-LASV toxicity study followed current FDA- and EMA-recognized guidelines and concepts for the nonclinical safety assessment of vaccines [35]. The study was conducted in compliance with Good Laboratory Practice (GLP) regulations with well-characterized EBS-LASV material. Exceptions to the GLP regulations included the analysis of virus shedding and ELISA testing, which were performed under non-GLP conditions at Auro Vaccines LLC using well-characterized assays according to established SOPs following Good Documentation Practices. The NZW rabbit was chosen as the animal model for this study, as it is an accepted non-rodent species for preclinical toxicity testing by regulatory agencies. Nonclinical vaccine safety assessments require the evaluation of potential toxicity in the presence of an active immune response. The EBS-LASV vaccine candidate induced a robust antibody response in the NSW rabbits, fulfilling this requirement.

The administration of 5.3 × 10^7^ PFU EBS-LASV (approximately five-fold higher than the highest proposed dose in humans) did not result in early mortality or any changes in clinical observations upon local irritation assessment, body temperature, body weight, food consumption monitoring, ocular assessments, as well as hematology, clinical chemistry, urinalysis, organ weights, or macroscopic and microscopic observations. Non-adverse transient increases in fibrinogen and CRP, indicative of an inflammatory reaction, were noted and were consistent with the pharmacology of EBS-LASV. Transiently increased fibrinogen and CRP post-vaccination are indicative of inflammation and/or an immune response, which is supported by the fact that all vaccinated animals seroconverted to EBS-LASV. No shedding of the EBS-LASV vector was detected in rabbit saliva or plasma samples, suggesting that secondary transmission of the EBS-LASV vaccine vector will not occur via saliva or blood in humans. The results of the EBS-LASV nonclinical repeat-dose toxicology were similar to nonclinical studies conducted for the licensed ERVEBO^®^ vaccine [11].

Other live-attenuated rVSV-vectored vaccines with similar rVSV backbones have been shown to be safe and immunogenic in various clinical trials. Previous human experience with an HIV-1 candidate vaccine (rVSV-N4CT1-HIVGag1) was reported by Fuchs. [10]. Safety monitoring included VSV cultures from blood, urine, saliva, and swabs of oral lesions; the local and systemic reactogenicity symptoms were mild to moderate and increased with the dose. No severe reactogenicity or serious product-related adverse events were reported, and all rVSV cultures were negative. Therefore, this attenuated replication-competent rVSV vaccine has an acceptable safety profile in healthy adults. Immunogenicity monitoring included the detection of VSV-neutralizing antibodies, HIV-1 specific binding antibodies, and T-cell immune responses; all vaccine recipients became seropositive for VSV after two vaccinations. Clarke [9] evaluated the safety and immunogenicity of a highly attenuated rVSV-based Ebola virus vaccine (rVSV-N4CT1-EBOVGP1). This vaccine was well tolerated at all dose levels tested and was immunogenic despite a high degree of attenuation.

The question of EBS-LASV vaccine efficacy in vivo has recently been addressed in the non-human primate (NHP) model [24], where it demonstrated immunogenicity and protection from a lethal LASV challenge after two IM doses of a quadrivalent vaccine containing a 1 × 10^7^ PFU EBS-LASV component. In addition, it was shown, in the same experiment, that a single dose of the quadrivalent vaccine containing 1 × 10^7^ PFU EBS-LASV was able to elicit serum LASV neutralizing antibodies. The second booster vaccination using the same quadrivalent vaccine markedly enhanced the antibody responses and was able to elicit serum LASV GP-specific IgGs and antigen-specific cellular responses (i.e., IFN-γ responses against LASV GP peptides).

The demonstration of EBS-LASV vaccine efficacy in the widely accepted NHP model for LASV disease and the nonclinical safety assessment, including the evaluation of neurovirulence, biodistribution, repeat-dose toxicity and virus shedding, supported the initiation of a Phase 1 randomized, placebo-controlled, dose-escalation study to evaluate the safety and immunogenicity of ESB-LASV in approximately 36 healthy adults at the Navrongo Health Research Centre and Kintampo Health Research Centre in Ghana [36]. The initiation of the clinical study represents a key milestone for providing a vaccine that can help protect vulnerable populations across West Africa and other regions against Lassa virus infection.

## Figures and Tables

**Table 1:** Study design and group designation for the GLP (N + 1) rabbit toxicity study.

Group	Test Material	Dose Route	Dose Volume (mL) ^1^	Dosing Days	Number of Rabbits ^2^			
					Main Study		Recovery Study	
					Males	Females	Males	Females
1	0.9% sodium chloride for injection, USP	IM	0.5	1, 22, 43	5	5	5	5
2	EBS-LASV (5.3 × 10^7^ PFU ^3^/rabbit)	IM	0.5	1, 22, 43	5	5	5	5

Abbreviations: USP, US Pharmacopeia; PFU, plaque-forming units; IM, intramuscular. ^1^ IM injection into the lumbar area (preferred) or lateral compartment of the thigh. ^2^ New Zealand White rabbits, approximately 2.8 to 3.9 kg and 4 months of age at the time of the first dose. ^3^ The target dose was 5 × 10^7^ PFU/0.5 mL; actual value administered was 5.3 × 10^7^ PFU/0.5 mL.

**Table 2:** Mouse neurovirulence study results.

Inoculum	IC Dose (PFU)	No. Surviving 24 h	No. Surviving 14 Days	% Survival
EBS-LASV	1 × 10^7^	10	10	100
	1 × 10^6^	10	10	100
	1 × 10^5^	10	10	100
	1 × 10^4^	10	10	100
	1 × 10^3^	10	10	100
rVSV-HIVGag5	1 × 10^2^	10	0	0
PBS	N/A	10	10	100

PBS, phosphate-buffered saline; IC, intracranial; PFU, plaque-forming units; N/A, not applicable.

**Table 3:** Rabbit body temperatures.

Timepoint (Study Day)	N Per Sex	Body Temp (°C)			
		Saline Control		EBS-LASV	
		Male	Female	Male	Female
1 (PR)	10	38.91 ± 0.22	39.02 ± 0.41	38.83 ± 0.27	38.95 ± 0.16
1 (2–4 HR)	10	38.73 ± 0.29	39.02 ± 0.20	38.98 ± 0.28	39.16 ± 0.18
22 (2–4 HR)	10	39.00 ± 0.35	39.05 ± 0.36	39.13 ± 0.14	39.51 ± 0.30 ^a^
43 (2–4 HR)	10	38.96 ± 0.37	39.28 ± 0.22	39.24 ± 0.32	39.31 ± 0.21
46	5	38.38 ± 0.33	38.38 ± 0.20	38.56 ± 0.40	38.70 ± 0.23
71	5	37.98 ± 0.43	38.68 ± 0.15	38.34 ± 0.34	38.86 ± 0.26

Values shown are the mean ± standard deviation; PR (pre-dose); 2–4 HR (two to four hours post-dose). ^a^ = *p* ≤ 0.01 versus Study Day 1 (PR) using Dunnett’s test. Study Days 46 and 71 body temperatures were measured during detailed examinations prior to euthanasia.

**Table 4:** Rabbit fibrinogen and CRP levels.

Timepoint (Study Day)	N Per Sex	Saline Control		EBS-LASV	
		Male	Female	Male	Female
Fibrinogen (mg/dL)					
−7	10	308.2 ± 26.7	358.2 ± 70.2	365.7 ± 80.3 ^b^	287.5 ± 36.4 ^b^
1 ^d^	10	291.3 ± 29.8	307.4 ± 37.6	325.3 ± 51.0	280.1 ± 58.4
3	10	308.0 ± 28.8	330.8 ± 66.3	402.4 ± 52.3 ^a^	332.7 ± 61.7
8	10	299.1 ± 30.6	329.3 ± 61.6	342.0 ± 54.2	302.3 ± 37.5
22 ^d^	10	234.4 ± 46.7	263.2 ± 23.3	268.3 ± 20.7	242.3 ± 24.8
24	10	265.9 ± 18.8	263.0 ± 37.1	344.1 ± 42.1 ^a^	262.1 ± 29.2
29	10	283.5 ± 50.6	272.7 ± 42.4	309.9 ± 26.8	229.8 ± 25.7 ^b^
43 ^d^	10	287.3 ± 37.5	247.8 ± 16.8	309.4 ± 37.5	214.9 ± 23.5 ^a^
45	10	290.7 ± 40.7	262.0 ± 14.9	345.9 ± 32.7 ^a^	262.1 ± 22.3
50	5	284.4 ± 28.1	253.3 ± 16.9	334.6 ± 14.8 ^a^	274.3 ± 13.0
71	5	281.6 ± 47.6	257.0 ± 23.9	284.0 ± 26.7	213.8 ± 20.7 ^b^
CRP (µg/mL)					
−7	10	2.47 ± 1.38	17.08 ± 23.38	3.81 ± 2.54	5.52 ± 3.98
2	10	4.73 ± 5.02	33.48 ± 30.96	60.07 ± 29.80 ^c^	89.27± 41.50 ^a^
3	10	2.67 ± 2.03	18.96 ± 22.65	28.90 ± 15.90 ^c^	31.18 ± 19.72
4	10	4.11 ± 1.73	33.46 ± 41.32	5.77 ± 3.16	7.28 ± 3.10
23	10	2.14 ± 1.13	10.71 ± 12.28	27.72 ± 20.38 ^c^	28.31 ± 16.95 ^b^
25	10	31.05 ± 78.84	6.98 ± 4.59	3.67 ± 2.89	3.19 ± 1.44 ^b^
44	10	1.98 ± 1.04	6.47 ± 4.43	14.12 ± 8.94 ^c^	22.47 ± 20.24 ^c^
46	10	2.78 ± 1.16	3.42 ± 1.83	2.71 ± 1.04	3.23 ± 1.80
71	5	2.06 ± 2.34	4.00 ± 3.47	0.98 ± 0.58	2.03 ± 1.24

^a^ = *p* ≤ 0.01, ^b^ = *p* ≤ 0.05 versus control using Dunnett’s test; ^c^ = *p* ≤ 0.01 versus control using Dunn’s test; ^d^ = day of dosing; male Group 1 (saline control) had an N = 9.

**Table 5:** Rabbit absolute organ weights.

Organ	Mean Absolute Organ Weights (g)			
	Saline Control		EBS-LASV	
	Male	Female	Male	Female
Study Day 46 (Main Study Group)				
Mean body weight (kg)	3.46 ± 0.21	3.22 ± 0.19	3.52 ± 0.18	3.80 ± 0.19 ^b^
brain	9.74 ± 0.38	9.69 ± 0.29	9.69 ± 0.37	10.04 ± 0.43 ^e^
epididymis	2.32 ± 0.45	N/A	2.56 ± 0.24	N/A
adrenal	0.40 ± 0.09	0.33 ± 0.10	0.45 ± 0.13	0.30 ± 0.06
pituitary	0.031 ± 0.005	0.029 ± 0.004	0.027 ± 0.003	0.045 ± 0.004 ^b,d^
prostate	0.62 ± 0.09	N/A	0.65 ± 0.10	N/A
thyroid	0.24 ± 0.05	0.25 ± 0.04	0.29 ± 0.02	0.34± 0.04 ^b^
heart	7.28 ± 0.63	6.01 ± 0.43	7.76 ± 0.74	6.48 ± 0.26
kidney	17.36 ± 1.55	13.90 ± 1.43	16.49 ± 1.20	16.36 ± 0.97 ^c^
liver	77.24 ± 8.87	55.16 ± 10.60	73.40 ± 6.50	71.52 ± 4.83 ^c^
lung	9.95 ± 0.72	10.23 ± 1.90	11.35 ± 3.26	10.73 ± 1.19
ovary	N/A	0.46 ± 0.25	N/A	0.29 ± 0.08
spleen	1.03 ± 0.07	1.48 ± 0.44	1.40 ± 0.27 ^a,e^	1.76 ± 0.46
testis	5.86 ± 1.02	N/A	6.23 ± 0.54	N/A
thymus	2.92 ± 0.70	2.89 ± 0.74	3.54 ± 0.55	3.68 ± 1.38
uterus	N/A	6.87 ± 2.64	N/A	7.49 ± 3.53
**Study Day 71 (Recovery Study Group)**				
Mean body weight (kg)	3.54 ± 0.17	3.54 ± 0.44	3.62 ± 0.11	4.14 ± 0.32 ^c^
brain	10.09 ± 0.48	9.76 ± 0.85	10.73 ± 0.28 ^c^	10.57 ± 0.47
epididymis	2.73 ± 0.39	N/A	2.98 ± 0.63	N/A
adrenal	0.45 ± 0.07	0.33 ± 0.04	0.47 ± 0.06	0.33 ± 0.05
pituitary	0.030 ± 0.000	0.038 ± 0.001	0.030 ± 0.002	0.041 ± 0.003
prostate	0.78 ± 0.19	N/A	0.70 ± 0.15	N/A
thyroid	0.33 ± 0.07	0.27 ± 0.04	0.27 ± 0.05	0.36 ± 0.06 ^c^
heart	7.74 ± 1.01	6.95 ± 0.61	7.96 ± 0.30	7.58 ± 0.44
kidney	15.46 ± 1.56	15.95 ± 2.94	16.91 ± 1.26	17.28 ± 2.01
liver	76.24 ± 23.96	63.08 ± 8.14	67.18 ± 4.45	71.99 ± 7.24
lung	12.27 ± 4.16	10.92 ± 1.10	10.63 ± 0.40	12.10 ± 2.20
ovary	N/A	0.25 ± 0.08	N/A	0.30 ± 0.08
spleen	1.21 ± 0.33	1.66 ± 0.62	1.47 ± 0.25	2.14 ± 0.66
testis	5.89 ± 0.46	N/A	6.38 ± 1.38	N/A
thymus	3.15 ± 0.86	3.53 ± 0.82	3.54 ± 0.81	6.08 ± 1.39 ^b,e^
uterus	N/A	6.21 ± 1.46	N/A	8.52 ± 2.10

^a^ = *p* ≤ 0.05 versus control using Wilcoxon test; ^b^ = *p* ≤ 0.01, ^c^ = *p* ≤ 0.05 versus control using *t*-test; ^d^ = *p* ≤ 0.01, ^e^ = *p* ≤ 0.05 versus control relative to body weight (*t*-test), N/A = not applicable.

## Data Availability

The data supporting this article will be made available by the authors on request.

## Supplementary Materials

The following supporting information can be downloaded https://www.mdpi.com/article/10.3390/vaccines12080858/s1, Table S1: Individual rabbit clinical observations for saline controls and EBS-LASV vaccinees; Table S2: Individual rabbit clinical chemistry values for saline controls and EBS-LASV vaccinees; Table S3: Historical pathology organ weight data for New Zealand white rabbits; Table S4: Summary of gross necropsy observations in rabbits in main study and recovery study groups.
