# Supplementary material for: Preclinical Safety Assessment of the EBS-LASV Vaccine Candidate against Lassa Fever Virus
Source: Vaccines (Basel). 2024 Jul 30;12(8):858. doi: 10.3390/vaccines12080858 (PMC11358935; doi:10.3390/vaccines12080858)
Supplement: Supplementary file 1 [file vaccines-12-00858-s001.zip › Table S3. Historical Pathology Organ Weight Data for New Zealand White Rabbits_v2.pdf]

Table S3. Historical Pathology Organ Weight Data for New Zealand White Rabbits

		Males				Females			
**Organ**	**Unit**	**N**	**Mean**	**S.D.**	**Range ***	**N**	**Mean**	**S.D.**	**Range ***
Bodyweight	Kg	18	3.3	0.4	2.4 - 3.8	18	3.5	0.7	2.1 - 4.4
Brain Weight	g	15	10.082	0.670	8.384 - 11.207	15	9.827	0.726	8.955 - 11.637
Epididymis Weight	g	15	2.609	0.617	1.788 - 3.740	N/A	N/A	N/A	N/A
Gland, Adrenal Weight	g	15	0.408	0.085	0.266 - 0.553	15	0.353	0.071	0.212 - 0.480
Gland, Pituitary Weight	g	15	0.027	0.009	0.009 - 0.044	15	0.036	0.014	0.013 - 0.072
Gland, Prostate Weight	g	15	1.069	0.710	0.335 - 2.822	N/A	N/A	N/A	N/A
Gland, Thyroid Weight	g	15	0.242	0.065	0.143 - 0.355	15	0.253	0.049	0.179 - 0.332
Heart Weight	g	15	7.632	0.661	6.547 - 9.129	15	6.920	0.732	5.438 - 7.692
Kidney Weight	g	15	15.860	1.668	12.351 - 19.650	15	15.388	1.940	12.182 - 18.878
Liver Weight	g	15	70.649	10.785	48.223 - 88.663	15	61.666	6.696	53.231 - 79.722
Lung Weight	g	15	11.394	1.966	9.200 - 17.489	15	10.393	0.808	8.708 - 11.718
Ovary Weight	g	N/A	N/A	N/A	N/A	15	0.428	0.150	0.232 - 0.698
Spleen Weight	g	15	1.350	0.367	0.723 - 2.157	15	1.857	0.551	1.201 - 3.093
Testis Weight	g	15	6.359	1.268	4.421 - 9.357	N/A	N/A	N/A	N/A
Thymus Weight	g	15	2.725	0.946	0.799 - 4.150	15	3.021	1.039	1.375 - 5.349
Uterus Weight	g	N/A	N/A	N/A	N/A	15	7.583	2.202	4.348 - 13.684

* N < 20: Lowest to highest; N > or equal 20: 2.5-97.5th percentile.
N/A = Non-Applicable
Age at necropsy for males: from 19 to 32 weeks old
Age at necropsy for females: From 19 to 32 weeks old
