# Supplementary material for: Preclinical Safety Assessment of the EBS-LASV Vaccine Candidate against Lassa Fever Virus
Source: Vaccines (Basel). 2024 Jul 30;12(8):858. doi: 10.3390/vaccines12080858 (PMC11358935; doi:10.3390/vaccines12080858)
Supplement: Supplementary file 1 [file vaccines-12-00858-s001.zip › Table S4. Summary of Gross Necropsy Observations in Rabbits in Main Study and Recovery Study Groups_v2.pdf]

Table S4. Summary of Gross Necropsy Observations for Male and Female Rabbits in Study Day 46 (Main Study Group) and Study Day 71 (Recovery Study Group)

	Study Day 46 (Main Study Group)				Study Day 71 (Recovery Study Group)			
	Male		Female		Male		Female	
	Saline Control	EBS-LASV	Saline Control	EBS-LASV	Saline Control	EBS-LASV	Saline Control	EBS-LASV
Number of Rabbits	5	5	5	5	5	5	5	5
**ARTERY, AORTA**								
Submitted	5	5	5	5	5	5	5	5
No Visible Lesions	5	5	5	5	5	5	5	5
**BONE MARROW, STERNUM**								
Submitted	5	5	5	5	5	5	5	5
No Visible Lesions	5	5	5	5	5	5	5	5
**BONE, FEMUR**								
Submitted	5	5	5	5	5	5	5	5
No Visible Lesions	5	5	5	5	5	5	5	5
**BONE, STERNUM**								
Submitted	5	5	5	5	5	5	5	5
No Visible Lesions	5	5	5	5	5	5	5	5
**BRAIN**								
Submitted	5	5	5	5	5	5	5	5
No Visible Lesions	5	5	5	5	5	5	5	5
**BRONCHUS**								
Submitted	5	5	5	5	5	5	5	5
No Visible Lesions	5	5	5	5	5	5	5	5
**CERVIX**								
Submitted	-	-	5	5	-	-	5	5
No Visible Lesions	-	-	5	5	-	-	4	4
Thick	-	-	0	0	-	-	1	1
**EPIDIDYMIS**								
Submitted	5	5	-	-	5	5	-	-
No Visible Lesions	5	5	-	-	5	5	-	-
**ESOPHAGUS**								
Submitted	5	5	5	5	5	5	5	5
No Visible Lesions	5	5	5	5	5	5	5	5
**EYE**								
Submitted	5	5	5	5	5	5	5	5
No Visible Lesions	5	5	5	5	5	5	5	5
**GALLBLADDER**								
Submitted	5	5	5	5	5	4	5	5
No Visible Lesions	5	5	5	5	5	4	5	5
Not Examined: Not Found at Necropsy.	0	0	0	0	0	1	0	0
**GALT**								
Submitted	5	5	5	5	5	5	5	5
No Visible Lesions	5	5	5	5	5	5	5	5
**GLAND, ADRENAL**								

	Study Day 46 (Main Study Group)				Study Day 71 (Recovery Study Group)			
	Male		Female		Male		Female	
	Saline Control	EBS-LASV	Saline Control	EBS-LASV	Saline Control	EBS-LASV	Saline Control	EBS-LASV
Submitted	5	5	5	5	5	5	5	5
No Visible Lesions	4	4	5	4	4	3	3	4
Focus, Raised	1	0	0	0	0	0	0	0
Enlargement	0	1	0	0	0	0	0	0
Focus, Dark	0	0	0	1	1	2	0	0
Nodule	0	0	0	0	0	0	2	1
**GLAND, HARDERIAN**								
Submitted	5	5	5	5	5	5	5	5
No Visible Lesions	5	5	5	5	5	5	5	5
**GLAND, LACRIMAL**								
Submitted	5	5	5	5	5	5	5	5
No Visible Lesions	5	5	5	5	5	5	5	5
**GLAND, MAMMARY**								
Submitted	5	5	5	5	5	5	5	5
No Visible Lesions	5	5	5	5	5	5	5	5
**GLAND, PARATHYROID**								
Submitted	5	5	5	5	5	5	5	5
No Visible Lesions	5	5	5	5	5	5	5	5
**GLAND, PITUITARY**								
Submitted	5	5	5	5	5	5	5	5
No Visible Lesions	5	5	5	5	4	5	5	5
Focus, Dark	0	0	0	0	1	0	0	0
**GLAND, PROSTATE**								
Submitted	5	5	-	-	5	5	-	-
No Visible Lesions	5	5	-	-	5	4	-	-
Focus, Dark	0	0	-	-	0	1	-	-
**GLAND, SALIVARY, MANDIBULAR**								
Submitted	5	5	5	5	5	5	5	5
No Visible Lesions	5	5	5	5	5	5	5	5
**GLAND, SALIVARY, PAROTID**								
Submitted	5	5	5	5	5	5	5	5
No Visible Lesions	5	5	5	5	5	5	5	5
**GLAND, SALIVARY, SUBLINGUAL**								
Submitted	5	5	5	5	5	5	5	5
No Visible Lesions	5	5	5	5	5	5	5	5
**GLAND, SEMINAL VESICLE**								
Submitted	5	5	-	-	5	5	-	-

	Study Day 46 (Main Study Group)				Study Day 71 (Recovery Study Group)			
	Male		Female		Male		Female	
	Saline Control	EBS-LASV	Saline Control	EBS-LASV	Saline Control	EBS-LASV	Saline Control	EBS-LASV
No Visible Lesions	5	5	-	-	5	5	-	-
**GLAND, THYROID**								
Submitted	5	5	5	5	5	5	5	5
No Visible Lesions	2	5	3	2	1	2	3	3
Small	1	0	0	0	0	0	0	0
Focus, Dark	2	0	2	2	4	3	2	1
Enlargement	0	0	0	1	0	0	0	0
Cyst, Pale	0	0	0	0	0	0	0	1
**HEART**								
Submitted	5	5	5	5	5	5	5	5
No Visible Lesions	5	5	5	5	5	5	5	5
**KIDNEY**								
Submitted	5	5	5	5	5	5	5	5
No Visible Lesions	3	3	5	5	5	5	5	5
Discoloration, Dark	2	2	0	0	0	0	0	0
**LARGE INTESTINE, APPENDIX**								
Submitted	5	5	5	5	5	5	5	5
No Visible Lesions	5	5	5	5	5	5	5	5
**LARGE INTESTINE, CECUM**								
Submitted	5	5	5	5	5	5	5	5
No Visible Lesions	5	5	5	5	5	5	5	5
**LARGE INTESTINE, COLON**								
Submitted	5	5	5	5	5	5	5	5
No Visible Lesions	5	5	5	5	5	5	5	5
**LARGE INTESTINE, RECTUM**								
Submitted	5	5	5	5	5	5	5	5
No Visible Lesions	5	5	5	5	5	5	5	5
**LARYNX**								
Submitted	5	5	5	5	5	5	5	5
No Visible Lesions	5	5	5	5	5	5	5	5
**LIVER**								
Submitted	5	5	5	5	5	5	5	5
No Visible Lesions	5	5	5	5	4	5	5	5
Discoloration, Pale	0	0	0	0	1	0	0	0
**LUNG**								
Submitted	5	5	5	5	5	5	5	5
No Visible Lesions	5	4	4	5	4	5	5	3

	Study Day 46 (Main Study Group)				Study Day 71 (Recovery Study Group)			
	Male		Female		Male		Female	
	Saline Control	EBS-LASV	Saline Control	EBS-LASV	Saline Control	EBS-LASV	Saline Control	EBS-LASV
Focus, Dark	0	1	1	0	1	0	0	2
**LYMPH NODE, ILIAC**								
Submitted	5	5	5	5	5	5	5	5
No Visible Lesions	5	5	5	5	3	4	5	5
Focus, Dark	0	0	0	0	2	0	0	0
Enlargement	0	0	0	0	0	1	0	0
**LYMPH NODE, MANDIBULAR**								
Submitted	5	5	5	5	5	5	5	5
No Visible Lesions	5	5	5	5	4	5	5	4
Focus, Dark	0	0	0	0	1	0	0	0
Enlargement	0	0	0	0	0	0	0	1
**LYMPH NODE, MESENTERIC**								
Submitted	5	5	5	5	5	5	5	5
No Visible Lesions	5	5	5	5	5	5	5	5
**MUSCLE, SKELETAL**								
Submitted	5	5	5	5	5	5	5	5
No Visible Lesions	5	5	5	5	5	5	5	5
**NERVE, OPTIC**								
Submitted	5	5	5	5	5	5	5	5
No Visible Lesions	5	5	5	5	5	5	5	5
**NERVE, SCIATIC**								
Submitted	5	5	5	5	5	5	5	5
No Visible Lesions	5	5	5	5	5	5	5	5
**OVARY**								
Submitted	-	-	5	5	-	-	5	5
No Visible Lesions	-	-	5	5	-	-	5	5
**OVIDUCT**								
Submitted	-	-	5	5	-	-	5	5
No Visible Lesions	-	-	5	5	-	-	2	3
Cyst, Pale	-	-	0	0	-	-	3	2
Mass	-	-	0	0	-	-	0	1
**PANCREAS**								
Submitted	5	5	5	5	5	5	5	5
No Visible Lesions	5	5	5	5	5	5	5	5
**SITE, INJECTION**								
Submitted	5	5	5	5	5	5	5	5
No Visible Lesions	4	4	4	5	5	5	5	5
Focus, dark	1	1	1	0	0	0	0	0

	Study Day 46 (Main Study Group)				Study Day 71 (Recovery Study Group)			
	Male		Female		Male		Female	
	Saline Control	EBS-LASV	Saline Control	EBS-LASV	Saline Control	EBS-LASV	Saline Control	EBS-LASV
**SKIN**								
Submitted	5	5	5	5	5	5	5	5
No Visible Lesions	5	5	5	5	5	4	5	5
Nodule	0	0	0	0	0	1	0	0
**SMALL INTESTINE, DUODENUM**								
Submitted	5	5	5	5	5	5	5	5
No Visible Lesions	5	5	5	5	5	5	5	5
**SMALL INTESTINE, ILEUM**								
Submitted	5	5	5	5	5	5	5	5
No Visible Lesions	5	5	5	5	5	5	5	5
**SMALL INTESTINE, JEJUNUM**								
Submitted	5	5	5	5	5	5	5	5
No Visible Lesions	5	5	5	5	5	5	5	5
**SMALL INTESTINE, SACCULUS ROTUNDUS**								
Submitted	5	5	5	5	5	5	5	5
No Visible Lesions	5	5	5	5	5	5	5	5
**SPINAL CORD, CERVICAL**								
Submitted	5	5	5	5	5	5	5	5
No Visible Lesions	5	5	5	5	5	5	5	5
**SPINAL CORD, LUMBAR**								
Submitted	5	5	5	5	5	5	5	5
No Visible Lesions	5	5	5	5	5	5	5	5
**SPINAL CORD, THORACIC**								
Submitted	5	5	5	5	5	5	5	5
No Visible Lesions	5	5	5	5	5	5	5	5
**SPLEEN**								
Submitted	5	5	5	5	5	5	5	5
No Visible Lesions	5	5	5	5	5	5	5	5
**STOMACH**								
Submitted	5	5	5	5	5	5	5	5
No Visible Lesions	3	2	4	3	5	4	5	5
Focus, Dark	2	3	1	2	0	0	0	0
Nodule	0	0	0	0	0	1	0	0
**TESTIS**								

	Study Day 46 (Main Study Group)				Study Day 71 (Recovery Study Group)			
	Male		Female		Male		Female	
	Saline Control	EBS-LASV	Saline Control	EBS-LASV	Saline Control	EBS-LASV	Saline Control	EBS-LASV
Submitted	5	5	-	-	5	5	-	-
No Visible Lesions	5	5	-	-	5	4	-	-
Small	0	0	-	-	0	1	-	-
**THYMUS**								
Submitted	5	5	5	5	5	5	5	5
No Visible Lesions	5	5	4	5	5	5	3	3
Focus, Dark	0	0	1	0	0	0	1	2
Nodule	0	0	0	0	0	0	1	0
**TONGUE**								
Submitted	5	5	5	5	5	5	5	5
No Visible Lesions	5	5	5	5	5	5	5	5
**TRACHEA**								
Submitted	5	5	5	5	5	5	5	5
No Visible Lesions	5	5	5	5	5	5	5	5
**URETER**								
Submitted	5	5	5	5	5	5	5	5
No Visible Lesions	5	5	5	5	5	5	5	5
**URINARY BLADDER**								
Submitted	5	5	5	5	5	5	5	5
No Visible Lesions	5	5	5	5	5	5	5	5
**UTERUS**								
Submitted	-	-	5	5	-	-	5	5
No Visible Lesions	-	-	5	5	-	-	4	4
Thick	-	-	0	0	-	-	1	1
**VAGINA**								
Submitted	-	-	5	5	-	-	5	5
No Visible Lesions	-	-	5	5	-	-	5	5
